# Preserving circumflex iliac lymph nodes to reduce the incidence of lower limb lymphedema following lymphadenectomy in cervical and endometrial cancers: A prospective randomized controlled trial

**DOI:** 10.1371/journal.pone.0311144

**Published:** 2024-12-02

**Authors:** Jianli Wang, Yan Lu, Fei Li, Desheng Yao

**Affiliations:** Department of Gynecologic Oncology, Guangxi Medical University Cancer Hospital, Nanning, Guangxi Zhuang Autonomous Region, P.R. China; IRCCS Burlo Garofolo Trieste, ITALY

## Abstract

Lower limb lymphedema (LLL) is a common postoperative complication following lymphadenectomy in cervical and endometrial cancers. Removal of the circumflex iliac nodes distal to the external iliac node (CINDEIN) is associated with LLL. Here, we sought to evaluate whether preserving the CINDEIN is helpful in reducing the incidence of LLL in women with cervical and endometrial cancers and to evaluate the safety of preserving CINDEIN. In this prospective randomized controlled trial, patients with clinical stage I A2 to II A cervical cancer and stage I to III endometrial carcinoma undergoing surgery were randomly assigned (1:1) to undergo pelvic lymphadenectomy with CINDEIN removal or preservation. The primary endpoint was the incidence of LLL at 24 months post-surgery. Eligible patients underwent sentinel lymph node (SLN) mapping with carbon nanoparticles (CNP). The study was registered with ClinicalTrials.gov, number ChiCTR2300071911. Between Jun 1, 2017, and Dec 31, 2018, 328 participants were randomly assigned to the two groups. Thirteen patients were excluded from the lymphedema analysis. A total of 158 patients in the CINDEIN preservation group and 157 in the CINDEIN removal group completed the follow-up examination. At baseline, no significant differences were observed between the two groups. The 3-year overall survival rate was 96.9% in the preservation group and 95.7% in the resection group. For cervical cancer and endometrial carcinoma, the incidence of LLL were significantly lower in the preservation group than in the removal group both at 24 months. No differences in the occurrence time of LLL were observed between the two groups. The LLL stages also showed no significant difference between the two groups. In the removal group, no CINDEIN metastases were identified in any patient. A total of 125 evaluable patients received the injection of CNP. None of the patients had CINDEIN detected as SLNs. CINDEIN removal is an important risk factor for LLL following lymphadenectomy in cervical and endometrial cancers. The metastasis rate of CINDEIN in cervical cancer and early endometrial cancer is relatively low, and preserving CINDEIN might be safe and helpful in reducing the occurrence of LLL.

## Introduction

Cervical cancer and endometrial cancer are the top two common gynecologic cancers, which were estimated to include 598,000 and 414,000 new cases in 2020, respectively [1]. Surgery is one of the main treatments in those patients. According to the National Comprehensive Cancer Network (NCCN) Guidelines, for International Federation of Gynecology and Obstetrics (FIGO) stages Ia2, Ib, and IIa cervical cancers and I ~ III endometrial carcinomas, pelvic lymphadenectomy (PLA) with or without (+/−) para-aortic lymphadenectomy (PALA) should be involved in surgical staging. However, the therapeutic benefit of lymphadenectomy is controversial because lymphadenectomy can increase the risk of intraoperative and postoperative complications such as bleeding, infections, organs injury, lymphedema, lymphocele, and lymphangitis. In particular, LLL has been shown to have a significantly negative impact on quality of life [2] since it is a chronic, irreversible, and generally incurable disease [3].

With regard to cervical and endometrial cancer, the extension of lymphadenectomy remains a topic of ongoing debate because the most frequently reported risk factors for LLL are post-operative radiotherapy [4, 5], high number of removed lymph nodes [6], and removal of circumflex iliac nodes distal to the external iliac node (CINDEIN) [7–9]. CINDEIN is a direct extension of the more distally located deep inguinal nodes or Cloquet’s node in the groin that drain the lower extremities [10]. Conventional pelvic lymphadenectomy includes removal of CINDEIN. A significant body of evidence were showed that removal of CINDEIN was independent risk factor for LLL and suggested that eliminating CINDEIN dissection from routine templates of bilateral pelvic lymphadenectomy could be helpful in reducing the incidence of LLL [7, 11, 12]. However, these previous studies had some limitations which might make it difficult to estimate the possible impact of CINDEIN removal on the incidence of LLL. First, they were all retrospective, observational designs. The selection bias could not avoid and some characteristics of the two study groups were unevenly distributed. Second, the studies may have diagnostic bias and the follow-up period varied greatly, which made it hard to identify the true incidence of LLL.

Considering that complete PLA increases organ injury, postoperative LLL and lymphocele [13–15], gynecological oncologists are beginning to use lymphatic mapping and SLN biopsy in cervical and endometrial cancer patients [16–18]. This technique greatly reduces the morbidities both intraoperative and postoperative [17, 18].

We conducted a prospective randomized controlled trial to compare the incidence of LLL between patients who underwent CINDEIN dissection after PLA and matched controls who did not undergo CINDEIN dissection. We also aimed to investigate the clinical significance of CINDEIN dissection from the following two aspects: the incidence of CINDEIN metastasis and the incidence of CINDEIN identified as SLN.

## Material and methods

### Study design and participants

This prospective, randomized trial was conducted at the Department of Gynecologic Oncology in Guangxi Medical University Cancer Hospital, Nanning, China, between June 1, 2017, and December 31, 2018. The inclusion criteria were age >18 years and International Federation of Gynecology and Obstetrics (FIGO) 2009 clinical stage IA2 to II A cervical cancer or I to III endometrial carcinoma. All eligible patients underwent radical or simple hysterectomy for PLA with or without para-aortic lymphadenectomy (PALA). Patients were excluded if they had a history of major lower extremity trauma or surgery, evidence of lower extremity thrombosis, primary lower limb lymphedema, or lower extremity oedema caused by cardiogenic or nephrogenic disease before surgery. This study was approved by the Research Ethics Committee of Guangxi Medical University Cancer Hospital, and all patients provided written informed consent.

This study followed the Consolidated Standards of Reporting Trials (CONSORT) reporting guideline, [19] which were recorded in detail in the CONSORT checklist.

### Randomization and masking

The patients were randomly assigned (1:1) to undergo PLA with CINDEIN removal or preservation. Randomization was performed using a computer-generated random number at the research center by the research assistant. The study was open-label, with both patients and investigators aware of treatment assignment.

### Sample size

The sample size amount was calculated considering that, as reported in the current literature [11], the occurrence rate of LLL in the CINDEIN dissection group and preserved group was 29.8% and 7.0%, respectively. Assuming an alpha-error of 5% with a power of 80%, the sample size was at least 43 women for each group. Considering a 10% rate of patient drop-out, a minimum amount of 48 patients for the group was considered necessary.

### Procedures

Patients were scheduled to have PLA with or without CINDEIN removal. Except for CINDEIN, seven pelvic lymph node sites were classified: common iliac nodes (CIN), external iliac nodes (EIN), internal iliac nodes (IIN), obturator nodes (ON), circumflex iliac nodes to distal obturator nodes (CINDON), parametrial nodes (PN), and cardinal ligament nodes (CLN). The definition of each site is as follows [20]: CIN are located between the level of the bifurcation of the common iliac artery and 2 cm above the bifurcation. EIN are located outside the lateral iliac artery and below the bifurcation of the common iliac artery. IIN are located on the anterior side of the medial iliac artery, below the level of the bifurcation of the common iliac artery between the two iliac arteries. ON are located on the anterior side of the obturator nerve and under the IIN, with CINDON being the most distal ON. PN are located along the uterine artery or vein, and CLN are located on the posterior side of the obturator nerve and are usually called deep ON. All surgical procedures were performed by the same surgeon.

We retrieved the clinical data of all the patients. Every participant was invited to attend a follow-up clinic appointment every 3 months including both objective (circumferential measurement) and subjective (Gynecologic Cancer Lymphedema Questionnaire, GCLQ) [21] evaluation tools for LLL diagnosis. Objective evaluation of LLL was mostly performed by comparative circumferential measurement, in which one limb was compared with the opposite at six specific points: the ankle joint, upper and lower border of the patella, 10cm above and below the patella, and 20cm above the patella. A circumferential difference of 2cm or more at the above levels between the two legs was consensual in diagnosing lymphedema. The presence of LLL was recorded during follow-up (at any timepoint after surgery) by means of the subjective judgment with Gynecologic Cancer Lymphedema Questionnaire (GCLQ) and objective measurement of the gynecologic oncologists. Once LLL was diagnosed in patient, the time from lymphadenectomy to symptom onset was evaluated, which was the definition of the time of LLL. For survival analysis, overall survival (OS) was defined as the time from randomization to death from any cause. All analyses were completed on the study database frozen on Dec 31, 2021.

### SLN navigation method

Among the patients enrolled in this study, 80 with cervical cancer and 45 with endometrial carcinoma who underwent laparoscopic surgery were subjected to SLN tracing using CNP. Briefly, for cervical cancer, the normal cervix submucosa, 0.2–0.5mm deep, around the tumor was injected with 1 ml of CNP at 3, 9 o’clock. While for endometrial carcinoma, cervical injection was performed by inserting a full needle (1 cm) at 3, 9 o’clock. The SLNs are identified at the time of surgery with direct visualization within 10 minutes after CNP cervical injection. All patients underwent SLN mapping using white light. The black dye results were recorded and SLNs were harvested. All the patients then underwent laparoscopic PLA.

### Outcomes

The primary outcome measure was the cumulative incidence of postoperative LLL. The cumulative incidence of LLL was compared between the two groups. The secondary outcome measures were the metastasis rate of circumflex iliac nodes, the overall survival rate at 3 years of follow-up and the rate of detection of CINDEIN as a SLN within 10 minutes after CNP cervical injection. The metastasis rate of circumflex iliac nodes and 3-year overall survival rate were compared between the two groups.

### Statistical analysis

Patient demographic and clinical characteristics were presented as mean (± standard deviation) for continuous variables and frequencies (%) for categorical variables. The t test was used to compare the two groups of continuous variables, and the chi-square test or Continuous correction chi-square test was used to compare the two groups of categorical variables. Kaplan-Meier method was used for survival analysis, and log-rank test was used to compare the survival rate of the two groups. SPSS version 23 (IBM Corp., Armonk, New York, USA) was used for statistical analysis. Statistical significance was set at P < 0.05.

## Results

**Fig 1:**
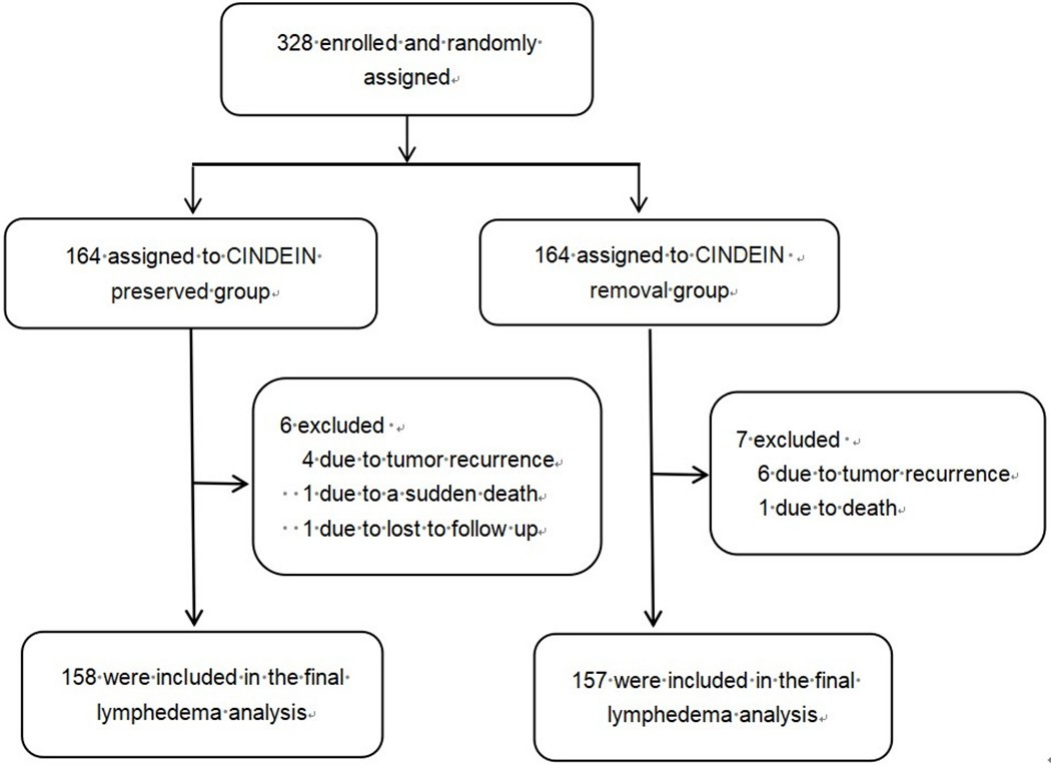
CONSORT flow diagram of the study.

From Jun 1, 2017 to Dec 31, 2018, a total of 328 patients were enrolled and randomly assigned to the two groups (164 in each group). The date of the first subject joining the group was June 3, 2017. 13 patients were excluded from the lymphedema analysis. Reasons for exclusion included a sudden death happened at 2 months after surgery (n = 1, in the CINDEIN preserved group), unable to contact (n = 1, in the CINDEIN preserved group), death because of thrombus (n = 1, in the CINDEIN removal group) and tumor recurrence (4 in the CINDEIN preserved group and 6 in the CINDEIN removal group). Lastly, 315 patients completed the lymphedema study (158 in the CINDEIN preserved group and 157 in the CINDEIN removal group, Fig 1) and were, thus, included in the modified intention-to-treat population (Table 1). Patient and disease baseline characteristics were well balanced between the two treatment groups (Table 1).

**Table 1:** Baseline characteristics.

	CINDEIN preserved group	CINDEIN removal group
**Age, years**	50.94±9.093	50.48±7.827
**Type of cancer**		
Cervical (%)	113 (71.5)	104 (66.2)
Endometrial (%)	45 (28.5)	53 (33.8)
**FIGO stage (2009)** **Of cervical cancer**		
I (%)	72 (63.7)	70 (67.3)
II (%)	41 (36.3)	34 (32.7)
**FIGO stage (2009)** **Of uterine cancer**		
I (%)	32 (71.1)	37 (69.8)
II (%)	4 (8.9)	5 (9.4)
III (%)	9 (20.0)	11 (20.8)
**High blood pressure**		
Yes (%)	11 (7.0)	18 (11.8)
No (%)	147 (93.0)	134 (88.2)
**LN metastasis**		
Positive (%)	31 (19.6)	25 (15.9)
Negative (%)	127 (80.4)	132 (84.1)
**Lymphocyst formation**		
Yes (%)	22 (18.0)	36 (25.9)
No (%)	100 (82.0)	103 (74.1)
**Adjuvant chemotherapy**		
Yes (%)	99 (62.7)	110 (70.1)
No (%)	59 (37.3)	47 (29.9)
**Adjuvant radiotherapy**		
Yes (%)	46 (29.1)	44 (28.0)
No (%)	112 (70.9)	113 (72.0)

CINDEIN, circumflex iliac nodes distal to the external iliac node; FIGO, International Federation of Gynecology and Obstetrics; LN, lymph nodes.

**Fig 2:**
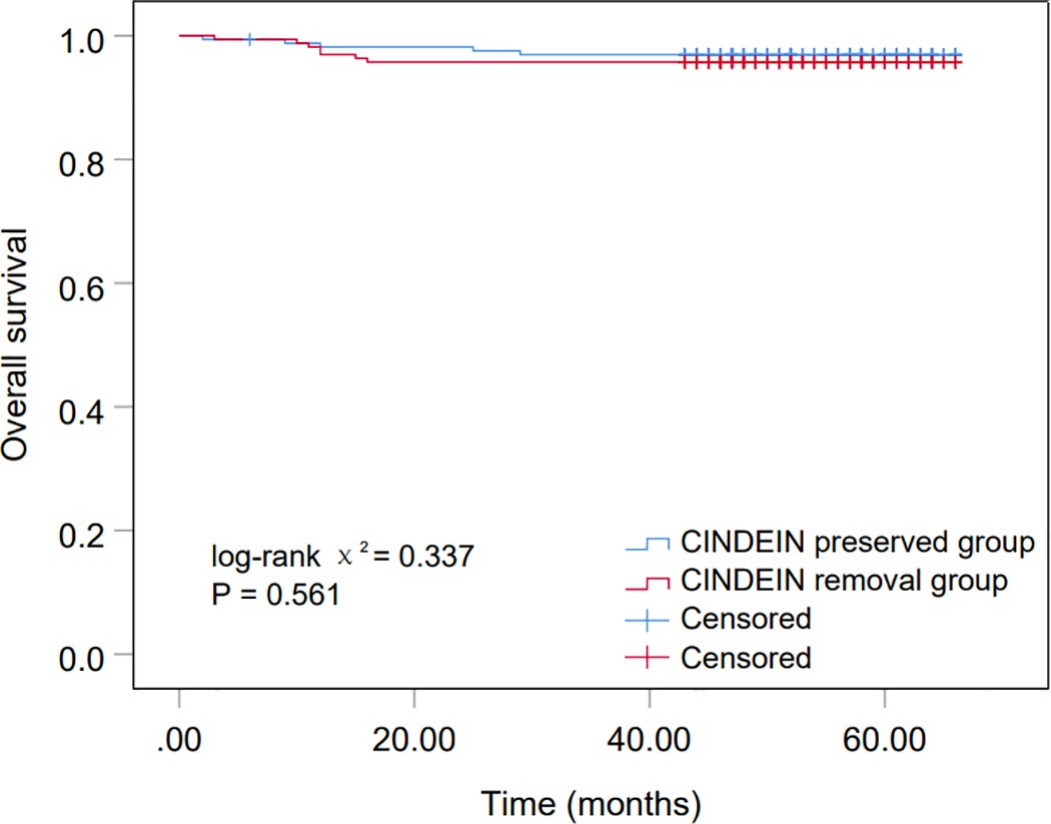
Survival analysis.

Five patients (3.0%) died in the CINDEIN preserved group (n = 164), of which 4 patients (2.4%) died of cancer. In the CINDEIN removal group (n = 164), 7 patients (4.3%) died, 6 of whom (3.7%) died of cancer. 3-year overall survival was 96.9% in the CINDEIN preserved group and 95.7% in the CINDEIN removal group. There was no significant difference in 3-year overall survival rate between the two groups. (log-rank χ^2^ = 0.337, p = 0.561; Fig 2).

For cervical cancer, the cumulative incidence of LLL at 24 months after surgery was significantly lower in the CINDEIN preserved group than in the CINDEIN removal group (p<0.001) (Table 2). For endometrial cancer, the cumulative incidence of LLL at 24 months after surgery was significantly lower in the CINDEIN preserved group than in the CINDEIN removal group (p = 0.048) (Table 3).

**Table 2:** Incidence of LLL between the CINDEIN preserved group and the removal group in the cervical cancer.

	CINDEIN preserved group	CINDEIN removal group	*P*
Cumulative incidence of LLL after 24 months of treatment (**%)**	5/117 (4.3%)	25/109 (22.9%)	<0.001*
**Time of onset of LLL**	14.00±6.52	8.19±4.75	0.053
**Stage of LLL**			
Stage I (%)	3 (60.00)	15 (60.00)	1
Stage II (%)	2 (40.00)	10 (40.00)	

Notes:

* means that the difference was statistically significant (P<0.05).

**Table 3:** Incidence of LLL between the CINDEIN preserved group and the removal group in the endometrial carcinoma.

	CINDEIN preserved group	CINDEIN removal group	*P*
Cumulative incidence of LLL after 24 months of treatment (**%)**	4/46 (8.7%)	14/54 (26%)	0.048*
**Time of onset of LLL**	9.50±7.33	7.36±3.23	0.393
**Stage of LLL**			
Stage I (%)	2 (50.00)	11 (78.60)	0.533
Stage II (%)	2 (50.00)	3 (21.40)	

Notes:

* means that the difference was statistically significant (P<0.05).

Both for cervical cancer and endometrial carcinoma, no difference in occurrence time of LLL was observed between the two groups (P = 0.053, P = 0.393, respectively). We also assessed the frequency of LLL and found no significant difference (P = 1, P = 0.533, respectively) between the two groups in stages 1 and 2 (Tables 2 and 3).

As a post hoc analysis, we assessed metastatic disease. In the removal group, 110 patients had stage IA2 to IIA2 cervical cancer and 54 had stage I to III endometrial carcinoma. No CINDEIN metastases were identified in any of the cases.

**Fig 3:**
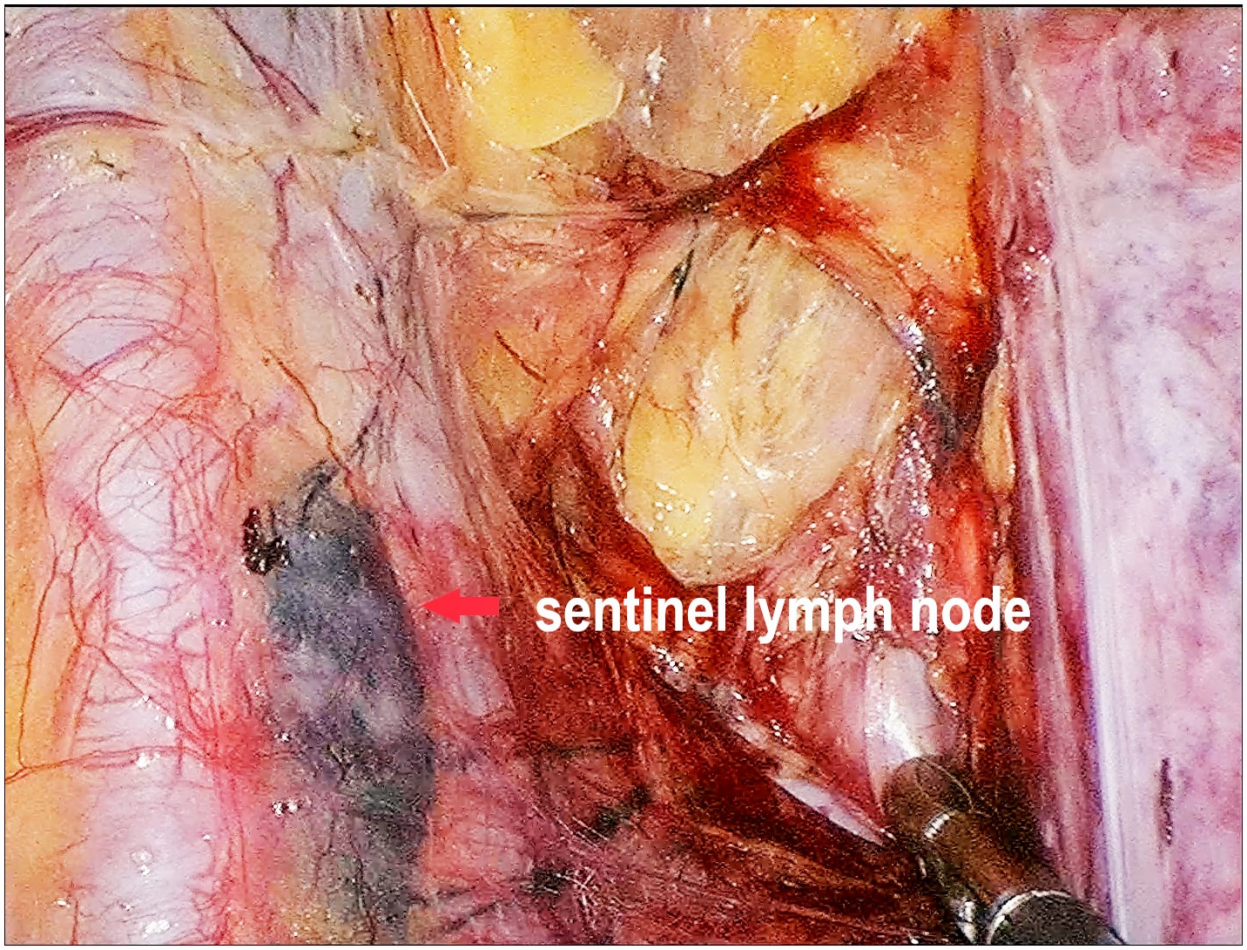
The application of CNP in SLN detection.

A total of 125 evaluable patients received the injection of CNP. In this cohort, at least one SLN was identified in 117 women (93.6%, Fig 3). A total of 210 SLNs were identified. Table 4 shows the results of SLN mapping. The most frequently detected SLN type was EIN (48.1%), followed by ON (25.9%). None of the patients had CINDEIN detected as SLNs. However, with prolonged operative time, CINDEIN appeared on black staining in five patients, while other pelvic lymph node sites were also developed.

**Table 4:** Detection of SLNs according to each subclassified site.

	Site						
	CINDEIN	EIN	IIN	ON	CIN	PN	CLN
**SLN**	0	102	30	55	18	3	2
**N (%)**	(0)	(48.1)	(14.2)	(25.9)	(8.5)	(1.4)	(0.9)

CINDEIN, circumflex iliac nodes distal to the external iliac node; EIN, external iliac nodes; IIN, internal iliac nodes; ON, obturator nodes; CIN, common iliac nodes; PN, parametrial nodes; CLN, cardinal ligament nodes.

## Discussion

LLL is a serious long-term lymphadenectomy complication that negatively affects a patient’s quality of life, and its incidence rate in cervical cancer may be as high as 55.9% [22], whereas, in endometrial carcinoma, the rate could be as high as 47% [23, 24]. Therefore, it is important to identify the risk factors for this complication. Several reports have shown that adjuvant pelvic radiotherapy [4, 5, 25], number of resected lymph nodes [7, 26], and excision of the circumflex iliac nodes are risk factors for the development of LLL, and CINDEIN removal is one of the significant risk factors [7, 11]. Unfortunately, as most studies were based on retrospective cohorts and without standardization of the methods used for lymphedema assessment, the reported incidence of LLL after CINDEIN removal varies [27]. A retrospective chart review showed that CINDEIN removal was not related to LLL but was associated with lymphoceles [8]. Moreover, almost half of the patients in previous reports received postoperative radiotherapy which also influenced the incidence of LLL [28].

In our study, selection bias was avoided because the baseline characteristics were similar between the CINDEIN preservation group and the removal group. No differences were identified in the clinical characteristics between the two groups, including postoperative radiotherapy. Both for cervical cancer and endometrial carcinoma, LLL were noted significantly more often after CINDEIN dissection than CINDEIN preservation at 24 months post-operatively. Although there were no differences noted in the time of lymphedema development, our exploratory findings demonstrated a trend towards delayed onset of lymphedema in the CINDEIN preservation group compared to the CINDEIN removal group. And our study did not show a significant difference in the stage of LLL between both treatment groups. A possible explanation for this discrepancy is the specific location of CINDEIN. CINDEIN is the most distal EIN, and the first intrapelvic lymph nodes to drain lymphatic fluid from the leg. A previous report showed that preserving CINDEIN was helpful for the formation of collateral pathways along the iliac and large abdominal vessels [29] which might explain the increased incidence of LLL and earlier occurrence of LLL when CINDEIN was removed. However, once LLL occurred, the removal of CINDEIN had no significant effect on the LLL stages. On the basis of these results, we believe that CINDEIN preservation may be useful for preventing LLL development.

Since pelvic lymph node metastasis is a major prognostic factor in patients with gynecologic malignancies, systematic lymphadenectomy is considered to be an important diagnostic and therapeutic tool. In our study, the 3-year overall survival rate was 96.9% in the preservation group and 95.7% in the resection group, which meant that whether the resection or preservation of CINDEIN had little effect on the 3-year overall survival rate. Both for patients with stage IA2 to II A cervical cancer and stage I to III endometrial carcinoma in our study, the metastatic rate of CINDEIN was 0%, similar to the findings of other retrospective studies [20, 30]. But in our clinical work, CINDEIN metastases might be found in non-endometrioid endometrial cancer patients with multiple pelvic lymph node metastases. It is possible that the origin of CINDEIN metastases might not be direct metastases from the primary tumor, they might occur after spread of the disease from other pelvic lymph nodes, such as ON, EIN, and IIN. CINDEIN metastases strongly correlated with multiple pelvic lymph node metastases. Moreover, CINDEIN were not the regional lymph nodes in early cervical cancer or endometrial carcinoma. No CINDEIN were identified as SLNs in this study. Similarly, several previous studies have reported that CINDEIN are seldom identified as SLNs in cervical cancer [20, 31, 32]. Although the nodal status is one of the most important prognostic factors for patients with cervical cancer and apparent early-stage endometrial cancer, the role of retroperitoneal staging in endometrial cancer is controversial [33]. Two independent randomized trials found that lymphadenectomy does not impact the progression free and overall survival but increases surgery-related morbidity [34, 35]. During the last decade, accumulating evidence suggests that sentinel node mapping could be a suitable alternative to lymphadenectomy. Ilaria Cuccu et al. reported that there was no difference in the 5-year disease-free and overall survival between sentinel node mapping and lymphadenectomy [36], similar to the findings of other retrospective studies [37–40]. Sentinel node mapping also does not increase the risk of nodal recurrence compared to lymphadenectomy in high-intermediate and high-risk endometrial cancer [36]. All these data supported the safety of adopting sentinel node mapping in endometrial cancer. Sentinel node mapping is not inferior to sentinel node mapping plus backup lymphadenectomy and conventional lymphadenectomy [37]. Therefore, it’s feasible to adopt sentinel node mapping plus backup lymphadenectomy which was performed in our study. We observed that no CINDEIN were identified as SLNs in endometrial carcinoma, which also indicated that CINDEIN were not the regional lymph nodes in endometrial carcinoma.

Based on these results, we suggest that if no obviously enlarged lymph nodes are found on preoperative imaging and intraoperative exploration, it is safe and feasible to preserve CINDEIN in patients with early cervical cancer and endometrial carcinoma. We believe that preservation of CINDEIN may be one of the best ways to reduce the incidence of LLL.

The strength of this study was its prospective randomized design. Our study had some limitations. The number of participants reported was not sufficiently large, and the efficacy estimates should be interpreted with caution. Second, our study was a single-center study, and future larger perspective multicenter studies are needed to confirm our findings.

## Conclusion

The removal of CINDEIN is an important risk factor for LLL in cervical cancer and endometrial carcinoma. The metastatic rate of CINDEIN in cervical cancer and early endometrial carcinoma is relatively low, and CINDEIN can be preserved to prevent the occurrence of LLL.

### Current trial status

This study was registered as ChiCTR2000040843 in the Chinese Clinical Trial Registry (https://www.chictr.org.cn).

## Supporting information

S1 Checklist Reporting checklist for randomised trial. (DOCX)

S1 Raw data (ZIP)

S1 File Trial study protocol. (DOCX)
