# Supplementary material for: Preserving circumflex iliac lymph nodes to reduce the incidence of lower limb lymphedema following lymphadenectomy in cervical and endometrial cancers: A prospective randomized controlled trial
Source: PLoS One. 2024 Dec 2;19(12):e0311144. doi: 10.1371/journal.pone.0311144 (PMC11611153; doi:10.1371/journal.pone.0311144)
Supplement: S1 File — (DOCX) [file pone.0311144.s003.docx]

**Trial study protocol**

1. **Statement**

## The research meets all applicable standards for the ethics of experimentation and research integrity. All data are reliability and validity. No potential conflict of interest is reported by the anthors and all the participants.

1. **Scientific title**

A prospective randomized controlled study on the relationship between preserving/removing circumflex iliac lymph nodes and lower limb lymphedema following lymphadenectomy in gynecological malignancies.

1. **Source of funding**

Self-financing.

1. **Research schedule**

2017.06—2018.12 To recruit participants and implement the trial intervention

2019.01—2021.12 To collect the clinical data of all the participants, the suvival and the incidence of Lower limb lymphedema will be followed up.

2022.01—2022.06 Statistical analysis

1. **Background**

Cervical cancer, endometrial cancer and ovarian cancer are are the top three gynaecologic cancer types, the incidences are increasing year by year. According to the National Comprehensive Cancer Network(NCCN) Guidelines, for International Federation of Gynecology and Obstetrics (FIGO) stages Ia2, Ib, and IIa cervical lesions, Ⅰ～Ⅲ endometrial cancers,and ovarian cancers, the surgical procedure involves pelvic lymphadenectomy (PLA) with or without (+/−) para-aortic lymphadenectomy (PALA), which is mainly performed for staging and treatment. Generally, systematic pelvic lymphadenectomy included resection of the common iliac nodes, external iliac nodes, internal iliac nodes, obturator nodes.However, the therapeutic benefit of lymphadenectomy is controversial because it can increase the risk of intraoperative and postoperative complications such as massive bleeding, nerve injury, lower limb lymphedema (LLL), pelvic lymphocele, and chylous ascites. In particular, LLL has been shown to have a significantly negative impact on quality of life. Severe LLL generally presents as a chronic, irreversible, and incurable disease.

As showed in previous studies and our studies, the frequently reported risk factors for LLL are post-operative radiotherapy, high number of removed lymph nodes, and removal of circumflex iliac nodes distal to the external iliac node (CINDEIN) and so on. A significant body of evidence suggests that eliminating CINDEIN dissection from routine templates of bilateral pelvic lymphadenectomy is helpful in reducing LLL.

CINDEIN are direct extensions of the more distally located deep inguinal nodes or Cloquet's node in the groin that drain the lower extremities. Since there are many adipose tissue around it, the CINDEIN are seemed to be large, which is easy to attract the attention of the operators and be resected during the operation. Conventional pelvic lymphadenectomy includes removal of CINDEIN. However, Previous studies showed that the metastatic rate of CINDEIN was low. It was reported that removal of CINDEIN was independent risk factors for LLL. A significant body of evidence suggests that eliminating CINDEIN dissection from routine templates of bilateral pelvic lymphadenectomy is helpful in reducing LLL.

However, previous studies used retrospective cohorts with limitations such as information bias or missing data, the short time of follow-up,which might make it difficult to estimate the possible impact of CINDEIN removal on the incidence of LLL. Here, we conduct a prospective randomised controlled trial to compare the incidence of LLL between patients who underwent CINDEIN dissection after pelvic lymphadenectomy and matched controls who did not undergo CINDEIN dissection. We also aim to investigate the clinical significance of CINDEIN dissection from the following two aspects: the incidence of CINDEIN metastasis and the incidence of CINDEIN identified as sentinel lymph nodes.

1. **Objective**

we sought to establish whether preserving the CINDEIN is helpful in reducing the incidence of LLL in women with cervical and endometrial cancers and to evaluate the safety of preserving CINDEIN.

1. **Eligibility criteria**

**Inclusion criteria:**

(1) Stages I b1~II A2 cervical cancer, I~III uterine malignant tumors, and various stages of ovarian malignant tumors undergoing satisfactory ovarian tumor cell reduction surgery with clear pathological diagnosis (FIGO 2009 staging);

(2) Age 18-75 years old;

(3) Willing to undergo surgery without any contraindications;

(4) All patient surgeries include pelvic lymph node dissection;

(5) Enrolled patients must understand and voluntarily participate in the study, and sign an informed consent form.

**Exclusion criteria:**

(1) Previous history of major lower limb trauma or surgery;

(2) evidence of lower extremity thrombosis, primary lower extremity lymphedema;

(3) Lower extremity edema caused by cardiogenic or nephrogenic diseases before surgery.

**8. Study design**

(1) This study will be a prospective, randomised trial carried out at the Department of Gynecologic Oncology in Guangxi Medical University Cancer Hospital, Nanning, China, between June 1, 2017, and December 31, 2018. Patients with indications for radical surgeries, including Stages I b1~II A2 cervical cancer, I~III uterine malignant tumors, and various stages of ovarian cancers will be enrolled in this study.

(2) The patients will be randomly assigned (1:1) to undergo pelvic lymphadenectomy with CINDEIN removal or preservation. Randomisation is performed using a computer-generated random number at the research centre by the research assistant.

① The clinical data of all the patients will be collected. Every participant will be invited to attend a follow-up clinic appointment every 3 months, and the study coordinator will phone each participant every 6 months after treatment to check for LLL. LLL is diagnosed using the Gynecologic Cancer Lymphedema Questionnaire (GCLQ). At the 3-month follow-up visit, the participants were examined by an experienced lymphedema specialist. Objective evaluation of LLL is mostly performed by comparative circumferential measurement, in which one limb is compared with the opposite at six specific points: the ankle joint, upper and lower border of the patella, ten above and below the patella, and 20 above the patella. A circumferential difference of 2 cm or more at several levels between the two legs is consensual in diagnosing lymphedema. Clinical information was collected until Dec 31, 2021. For survival analysis, progression-free survival (PFS) and overall survival (OS) are defined as the time between enrolment and disease progression or death, respectively.

② Among the patients enrolled in this study, patients with cervical cancer and endometrial carcinoma who underwent laparoscopic surgery will be subjected to sentinel lymph node (SLN) tracing using carbon nanoparticles (CNP). The black dye results will be recorded and SLNs will be harvested.

**9. Sample size**

The sample size amount was calculated considering that, as reported in the current literature,11 the occurrence rate of LLL in the CINDEIN dissection group and preserved group was 29.8% and 7.0%, respectively. Assuming an alpha-error of 5% with a power of 80%, the sample size was at least 43 women for each group. Considering a 10% rate of patient drop-out, a minimum amount of 48 patients for the group was considered necessary.

1. **Randomization Procedure**

Random Number Generators.

1. **Outcomes**

Primary indicator: the incidence of Lower limb lymphedema.

Secondary indicator: the metastasis rate of circumflex iliac nodes.

1. **The definition of loss to follow-up**

The researchers are unable to get the final observation results since the participant could not be contacted due to the change of phone numbers.

1. **Management of adverse events**

It is considered that this study does not increase any specifc risk for the participants beyond those surgery in general. All adverse events will be recorded, including those spontaneously reported by participants and observed by investigators, and the relevance to the study will be identifed. The researchers will immediately take appropriate medical measures to protect the rights of the subjects.

1. **Research ethics approval**

This study will be approved by the Research Ethics Committee of Guangxi Medical University Cancer Hospital, and all patients provided written informed consent.

1. **Recruitment**

The participants will be enrolled at the Department of Gynecologic Oncology in Guangxi Medical University Cancer Hospital, Nanning, China by two qualified and experienced gynecologic oncologists. The testing subjects participating the clinical trials are voluntary.

1. **Data collection**

Baseline characteristics of the participants will be collected, including age, history of high blood pressure, type of cancer, FIGO stage, LN metastasis, lymphocyst formation, adjuvant chemotherapy or adjuvant radiotherapy, the metastasis rate of circumflex iliac nodes and the survival analysis.

1. **Statistical analysis**

All continuous variables are described using means and standard deviations (SDs). Categorical variables are reported as percentage values, and statistical tests such as the Shapiro-Wilk (S-W) test, chi-square test, or Fischer’s exact test were used to analyse the primary and secondary study objectives. The independent prognostic significance was calculated using logistic regression analysis. SPSS version 23 (IBM Corp., Armonk, New York, USA) was used for statistical analysis. Statistical significance was set at P < 0.05.

1. **Participant management**
2. The testing subjects participating the clinical trials are voluntary and may withdraw from the study for any reason at any time.
3. The privacy of the subjects will be protected and the information collected during the study are also kept confidential.
4. **Management of collecting sample**
5. The collection, testing, transportation, preservation and destruction of biological samples related to the research should strictly abide by China's current effective laws and regulations, as well as the relevant quality control standards confirmed by both hospitals and central laboratories.
6. We ensure that biological samples and their data should not be used for any other purpose that has nothing to do with this study.
7. **Drug management**

All the drugs used in the research will be provided by the pharmacy department of hospital, and the storage, distribution, verification and use are carried out in accordance with the standards.

1. **Data management**

The research data will be collected through the hospital medical record information system, and the study coordinator phoned each participant every 6 months after treatment to check for LLL.

1. **Ancillary and post-trial care**

If the subjects experiences disease progression, active medical measures will be given.
